# Engineered exosomes deliver structurally optimized toad BAX to reactivate mitochondrial apoptosis in colorectal cancer

**DOI:** 10.3389/fonc.2026.1882315

**Published:** 2026-06-24

**Authors:** Xinqiang Xu, Hongjie Wu, Yixin Yan, Hongwei Cui, Tianyi Yu, Ye Yang, Zhendong Deng, Jinjun Qian

**Affiliations:** 1 Nanjing Hospital of Chinese Medicine Affiliated to Nanjing University of Chinese Medicine, Nanjing, China; 2 School of Medicine, Nanjing University of Chinese Medicine, Nanjing, China; 3 School of Elderly Care Services and Management, Nanjing University of Chinese Medicine, Nanjing, China

**Keywords:** BAX, BCL2, colorectal cancer, mitochondrial apoptosis, structural optimization, targeted delivery

## Abstract

**Introduction:**

Mitochondrial apoptosis evasion, driven by overexpression of anti-apoptotic BCL-2 family proteins, remains a major obstacle in the effective treatment of colorectal cancer (CRC). While BCL-2 homology 3 (BH3) mimetics (such as venetoclax) have shown clinical efficacy in hematologic malignancies, their effectiveness in solid tumors is limited. Direct delivery of pro-apoptotic effectors, including BAX, offers an alternative strategy; yet, the therapeutic potential of non-human BAX orthologs and their delivery methods has not been explored.

**Methods:**

The functional activity of Xenopus laevis (African clawed frog) BAX was evaluated in human CRC cell lines (HCT116, LOVO) and in cell line-derived xenograft (CDX) and AOM/DSS-induced CRC mouse models. Direct binding to human BCL-2 was quantified by microscale thermophoresis. The mechanism of action was elucidated via JC-10 staining, subcellular fractionation and liposome permeabilization assays.Structure-guided mutagenesis based on the BCL-2–Beclin-1 BH3 complex (PDB: 5VAU) was employed to generate a triple mutant (I86T, A102S, R109M) . Optimized BAX was packaged into engineered exosomes for targeted delivery, and their anti-tumor efficacy and safety were assessed in CDX, patient-derived xenograft (PDX), and AOM/DSS models.

**Results:**

Toad BAX directly bound human BCL-2 (Kd = 12.7 ± 1.9 µM), triggered mitochondrial outer membrane permeabilization (MOMP), cytochrome c release, and caspase-9/3 activation, thereby suppressing CRC cell proliferation and inducing apoptosis. The rationally designed triple mutant exhibited enhanced BCL-2 affinity and superior in vivo antitumor activity compared to wild-type toad BAX. Exosome-mediated delivery of the optimized BAX efficiently targeted CRC cells, inhibited tumor growth in PDX models, and extended overall survival in AOM/DSS-induced CRC without inducing overt toxicity.

**Discussion:**

This study establishes structurally optimized cross-species BAX, delivered via engineered exosomes, as a potent and safe strategy to reactivate mitochondrial apoptosis against CRC. It provides a preclinical foundation for protein-based therapeutics targeting apoptosis-evasive solid tumors, offering a mechanistically distinct alternative to conventional BH3 mimetics.

## Graphical Abstract

**Figure:**
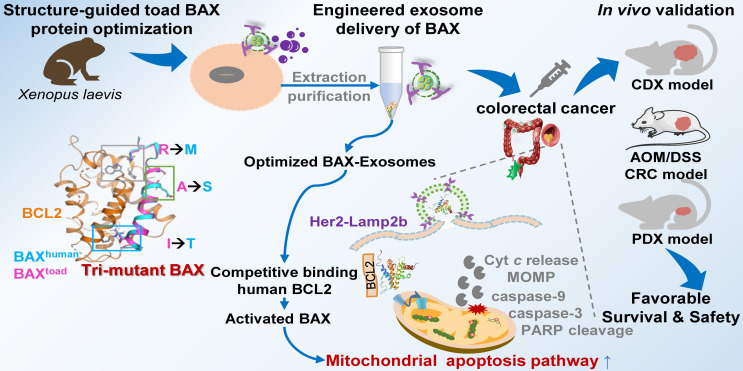
This study establishes structurally optimized cross-species BAX, delivered via engineered exosomes for targeted induction of mitochondrial apoptosis in colorectal cancer (CRC). Based on an evolution-inspired comparative optimization of the BAX protein from Xenopus laevis, the structure-guided triple mutations (I86T, A102S, R109M) within the BH3 domain were introduced to enhance binding affinity to human BCL2, thus reactivating mitochondrial apoptosis. The optimized tri-mutant BAX was packaged into tumor-targeted extracellular vesicles (EVs) displaying Her2-Lamp2b for CRC-specific delivery. Upon cellular internalization, BAX competitively neutralizes BCL2, triggering mitochondrial outer membrane permeabilization (MOMP), cytochrome c release, caspase-9/3 activation, and PARP cleavage. Systemic administration of BAX-EVs suppressed tumor growth and extended survival in cell-derived xenograft (CDX), AOM/DSS-induced CRC, and patient-derived xenograft models (PDX) without systemic toxicity. This potent and safe pro-apoptotic strategy may effectively overcome BCL2-mediated apoptosis resistance, paving the way for a translationally viable anti-CRC therapy.

## 1 Introduction

Despite advances in surgical techniques, chemotherapy, and targeted therapies, colorectal cancer (CRC) remains the third most common cancer in global incidence and the second leading cause of cancer-related mortality, largely due to toxicity and intrinsic/acquired resistance to conventional therapies (1, 2). A hallmark underlying CRC progression and therapeutic resistance is the evasion of programmed cell death, where dysregulation of the intrinsic mitochondrial apoptosis pathway is a core mechanism by which tumor cells evade programmed cell death (3). The B cell lymphoma 2 (BCL2) protein family governs the intrinsic apoptotic pathway and regulates mitochondrial outer membrane permeabilization (MOMP) via the pro-apoptotic effectors BAX (BCL2-associated X protein)/BAK, as essential executioners (4). Upon activation, BAX undergoes a conformational change, translocates to the mitochondria, and oligomerizes to form pores that release cytochrome *c* (Cyt *c*), thereby activating caspase-9 and the effector caspase cascade (5). The anti-apoptotic members such as BCL2, BCL-XL, and MCL-1 sequester BAX via their hydrophobic homology 3 (BH3) domain-binding groove, thus maintaining a delicate equilibrium that determines cellular fate (4, 6).

Given their pivotal roles, pharmacological strategies to activate BAX or neutralize BCL2 have been developed and shown promise, including several BH3 mimetic peptides and small molecules (7). Numerous natural products (such as resveratrol and oridonin) have been reported to modulate the BAX/BCL2 ratio and induce mitochondrial apoptosis in CRC cells (8); The first FDA-approved BH3 mimetic, venetoclax (ABT-199), which displaces sequestered BH3-only proteins, has revolutionized the treatment of hematologic malignancies, but has shown limited monotherapy efficacy in solid tumors including CRC, partly due to compensatory upregulation of MCL-1 and BCL-XL, as well as intrinsic resistance mechanisms (9, 10). However, these agents are constrained by poor bioavailability, off-target toxicity, and/or a lack of direct BAX activation specificity. Thus, direct delivery of the effector protein BAX, bypassing upstream regulation to execute the terminal step of apoptosis, represents a conceptually attractive alternative. A major barrier to protein−based therapeutics is the lack of efficient and safe delivery, including poor membrane penetration, rapid proteasomal degradation, and inability to reach intracellular targets (7). Recently, engineered extracellular vesicles (EVs or exosomes) have emerged as a superior delivery platform due to their ability to target tumors, evade immune surveillance, and protect cargo from degradation (11).

Despite the therapeutic potential of BAX activation, no study has systematically evaluated cross-species BAX variants or biochemical properties that could be amenable to therapeutic exploitation. Interestingly, BAX orthologs are highly conserved across vertebrates, yet subtle sequence variations may harbor distinct biochemical properties. For example, BAX from *Xenopus laevis* (African clawed frog) shares ~70% amino acid identity with human BAX but exhibits distinct surface charge distributions that may alter its binding affinity for and interactions with human anti−apoptotic BCL2. Whether such cross−species BAX retains robust pro−apoptotic activity in human CRC cells, and whether it can be exploited therapeutically remains unexplored. Deciphering these features could provide both evolutionary insights and structural clues for optimizing pro-apoptotic protein-based anticancer agents.

Based on the established model that BCL2-like proteins intercept apoptotic signals by sequestering activated BAX, exogenous BAX overexpression may function as a competitive inhibitor that overwhelms endogenous anti-apoptotic buffering capacity (3). We thus hypothesized that toad-derived BAX from *Xenopus laevis* could restrict CRC progression by competitively binding human BCL2 to induce mitochondrial apoptosis. Here, we investigated the therapeutic potential of toad BAX in CRC and demonstrated that it effectively suppressed proliferation and induced mitochondrial apoptosis *in vitro* and *in vivo*. Mechanistically, BAX binds directly to human BCL2, displacing it from pro−survival complexes and triggering MOMP. Through structure−guided mutagenesis based on the BCL-2-Beclin-1 complex aimed to test whether rationally engineering the BH3 interface of a cross-species BAX homolog could enhance its binding to human BCL-2 to a level comparable to that of human BAX, we optimized a triple mutant BAX to enhance its binding affinity for BCL2 as a generalizable protein engineering pipeline, thereby improving its anti-tumor activity. Finally, we also developed a targeted delivery system to package BAX into exosomes and deliver it to CRC sites, and validated its therapeutic efficacy and safety in multiple CRC models. Our findings establish cross−species BAX as a viable therapeutic modality and highlight rational protein engineering combined with exosomal delivery as a powerful strategy of precise targeted therapy against CRC.

## 2 Materials and methods

### 2.1 Antibodies and reagents

The primary antibodies were BAX (2774s, Cell Signaling Technology), PARP (9542S, Cell Signaling Technology), Cleaved Caspase 3 (9661S, Cell Signaling Technology), HA (51064-2AP, ProteinTech Group, China), GAPDH (60004-1-IG, ProteinTech Group), Alix (2171S, Cell Signaling Technology), CD9 (13174S, Cell Signaling Technology), Cytochrome C (A13430, Abclonal), calnexin (10427-2-AP ProteinTech Group), β-actin (66009-1-Ig, ProteinTech Group), COX4 (bsm-33037M, Bioss), caspase 9 (10380-1-AP, ProteinTech Group), DYKDDDDK (also called FLAG; 14793S, Cell Signaling Technology). The second antibodies were goat anti-Rabbit IgG(H+L) HRP (FMS Rb01, Fcmacs) or goat anti-mouse (S0002, Affinity) at the dilutions of 1:5000. Trizol reagent (10606ES60), Hifair 1st Strand cDNA Synthesis SuperMix for qPCR (gDNA digesterplus) (11121ES60), and SYBR Green PCR master mix (11201ES03) were purchased from Yeasen Biotechnology (Shanghai) Co., Ltd.

### 2.2 Cell lines and cell culture

HCT116 and LOVO cells were cultured in RPMI 1640, and HEK293 cells were cultured in DMEM, with 10% fetal bovine serum, 100 U/mL penicillin, and 100 μg/mL streptomycin. The cells were cultured at 37 °C in 5% CO_2_.

### 2.3 Plasmids and transfection

The full-length *Xenopus laevis BAX* (NM_001085635) linked with a HA tag was cloned into PLC5-ciR (GS0104, Guangzhou Geneseed Biotech Co, China) between *Eco*RI and *Bam*HI sites. Site-mutations of (I86T/A102S/R109M) were constructed via overlap PCR and Mut Express II Fast Mutagenesis Kit V2. The lentivirus plasmid pLV3-CMV-BAX(human)-3×FLAG-Puro expressing human *BAX* (NM_001291428.2) was ordered from MiaolingBio (P62706, Wuhan, China).

### 2.4 Cell proliferation, colony formation, and cell apoptosis assays

Cell proliferation, colony formation, and apoptosis were assessed using CCK8, colony formation, and Annexin-V/PI staining, as previously described (12).

### 2.5 Transfection and preparation of EVs

HEK293 cells were co-transfected with BAX lentivirus and Her2/Lamp2b (Human epidermal growth factor receptor 2/Lysosome-associated membrane protein 2b) lentivirus to generate HEK293 *BAX*-OE (overexpression) cells, respectively. After 48 h of transfection, puromycin selection was used to isolate stably transfected cells. Exosomes containing BAX (BAX-EVs) were isolated from the supernatants of HEK293 Her2/Lamp2b *BAX*-OE and HEK293 *BAX*-OE. We purified BAX-EVs using a series of centrifugation steps (300×g to remove cells, 10,000×g to remove shedding vesicles, 3,000×g to remove any remaining cell debris). The supernatant was filtered through a 0.22 μm filter and subjected to ultracentrifugation at 200,000×g for 90 min. The resulting pellets were resuspended in PBS and purified through additional ultracentrifugation at 200,000×g.

### 2.6 Characterization of EVs

Western blotting (WB) was used to analyze the expression of Alix and CD9, two marker proteins of exosomes, in purified EVs. The morphology of the EVs was characterized using a JEOL 2100 transmission electron microscope. Nanoparticle tracking analysis (NTA) was employed to determine the size distribution of the particles.

### 2.7 Immunofluorescence staining and confocal imaging

JC-10 dye was added to the cells according to the manufacturer’s instructions (C2004, Beyotime, China). Cells were plated in confocal dishes and allowed to adhere overnight. The culture medium was then removed, and JC-10 dye was added to a final concentration of 5 μM. Following a 30 min incubation at 37 °C, the cells were gently washed with PBS and 1 mL PBS was added to maintain humidity. Images were captured using a confocal microscope (TCS SP8, Leica, Germany) with excitation/emission settings of 490/525 nm for monomers and 540/590 nm for J-aggregates.

### 2.8 Establishment of the subcutaneous xenograft model

HCT116 EV and *BAX*-OE cells (1 × 10^6^) were subcutaneously injected into the bilateral flanks of 6-8-week-old nude mice. Tumor growth was monitored every 2 days, and tumor volume was calculated according to the formula: length × width^2^/2. Tumors were harvested for photographing and weighed once they had reached a diameter of 15 mm.

### 2.9 The azoxymethane/dextran sulfate sodium-induced CRC model

Empty vector (EV) and AAV9-loaded *BAX*-OE vector were purchased from GeneChem, Inc. Tail vein injections were carried out as follows: 1 × 10^11^ virus genome (vg) of AAV9 in 100 μL of saline was injected into the tail veins of 4-week-old BALB/c. C57BL/6J mice (6–8 weeks old, with equal numbers of males and females) were randomly divided into 2 groups (n = 10). The transduction efficiency of AAV9 was evaluated using WB assay. The mice received injections of 10 mg/kg AOM (*i.p.*) and then were subjected to 3 cycles of 1-week oral administration of 2.5% DSS followed by 2 weeks of regular drinking water. Survival and body weight were recorded throughout the experiment.

### 2.10 Intravenous delivery of BAX-EVs *in vivo*

BAX-EVs (5 mg/kg) were administered intravenously every other day, respectively.

### 2.11 Preparation of liposomes

Liposomes of MOM lipid composition and with cascade blue (CB) encapsulated were prepared by the extrusion method. Plain liposomes, lacking fluorophores, were prepared similarly, except that buffer A used for lipid resuspension contained no fluorophores, and gel filtration chromatography was omitted post-extrusion. Liposomes containing lipophilic quenchers were prepared with the same method.

### 2.12 Assessment of cascade blue release from liposomes by fluorescence quenching

Liposomes (50 μM) with CB dyes encapsulated were mixed with 2 μg/mL anti-CB antibodies in 250 μL of buffer. The initial emission intensity (F0) was determined after 5 min of equilibration at 25 °C. Purified BAX_234aa protein was then added. The first fluorescence intensity measurement was started precisely 20 s after adding the protein and was followed by multiple measurements at predetermined time intervals for 2 h, resulting in multiple intermediate intensities (F).

### 2.13 Statistical analyses

Statistical analyses were performed using SPSS version 22.0, and all values were expressed as mean ± standard deviation (SD) unless otherwise specified. A two-tailed Student’s t-test (2 groups) or one-way analysis of variance (ANOVA) (≥3 groups) was utilized to evaluate statistical significance. *P* < 0.05 was considered statistically significant. Significance levels were denoted as *P for *P* < 0.05, **P for *P* < 0.01, ***P for *P* < 0.001, and n.s. for no significance.

## 3 Results

### 3.1 Toad-derived BAX exerts anti-proliferative and pro-apoptic effects in CRC

**Figure 1:**
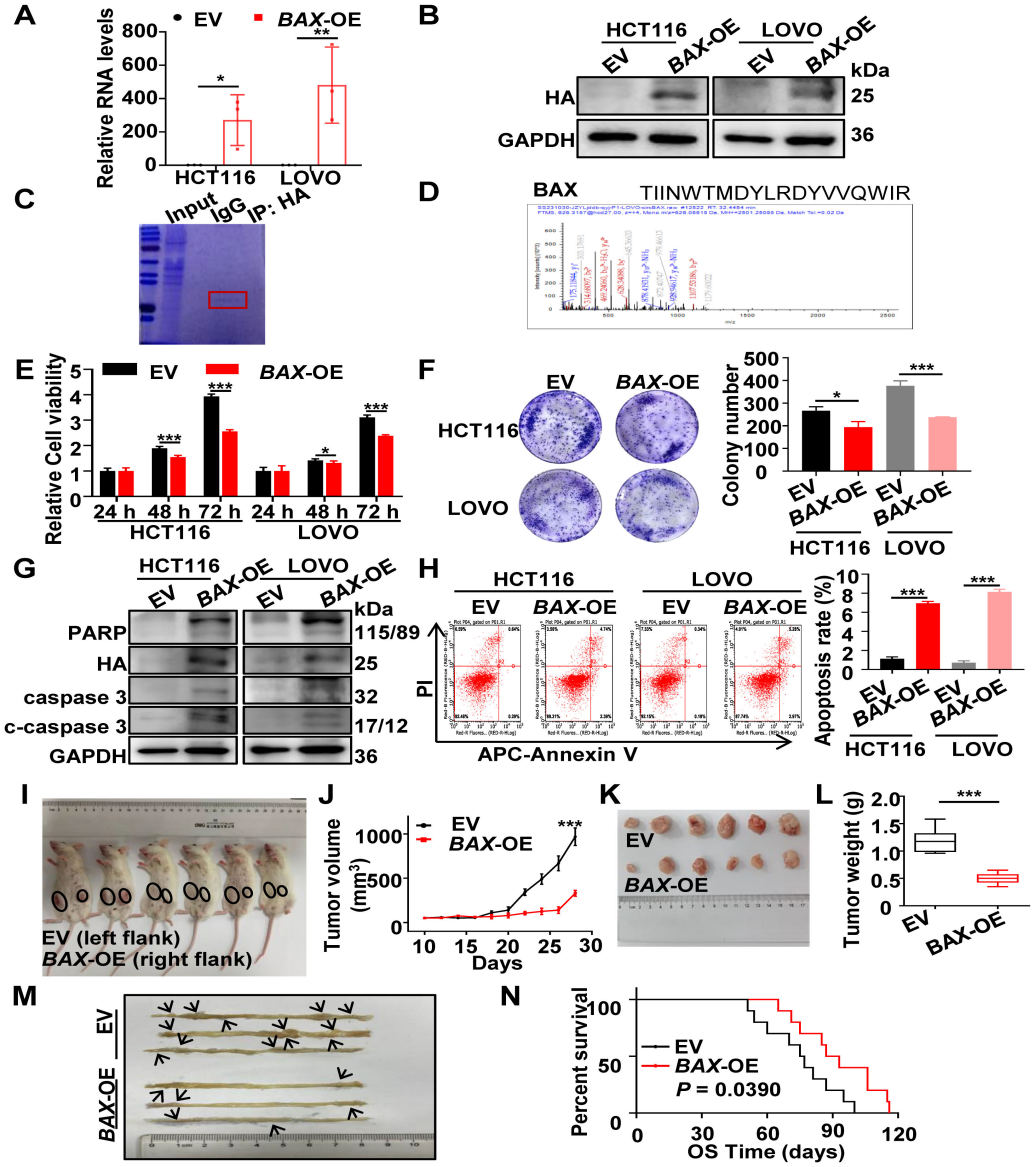
Toad-derived BAX inhibits CRC cellular proliferation and promotes apoptosis *in vitro* and *in vivo*. **(A)** Overexpression of *Bax* mRNA (NM_001085635) from *Xenopus laevis* (African clawed frog) in HCT116 and LOVO cells was determined by RT-qPCR. **(B)** WB analysis confirmed overexpression of HA-tagged BAX_234aa in HCT116 and LOVO cells. **(C)** Coomassie Brilliant Blue staining of the gel showed the HA-antibody-enriched BAX_234aa protein band with the expected size. **(D)** Mass spectrometry analysis identified specific peptides from BAX. **(E)** Overexpression of toad-derived BAX_234aa resulted in a lower cell proliferation rate in HCT116 and LOVO cells, as measured by CCK8 assays. **(F)** Representative images from a colony-formation assay showed fewer colonies formed by *BAX*-OE cells compared to control cells. **(G)** WB analysis demonstrated increased BAX-induced apoptosis, as indicated by cleavage of PARP and caspase 3. **(H)** Overexpression of BAX induced cell apoptosis, as quantified by APC-Annexin V staining. **(I)** Photographic images of CDX mice on day 28 and tumors taken from the mice in each group (n = 6). **(J)** Tumor growth curves of the CDX mice in the Ctrl and *BAX*-OE groups. **(K-L)** Tumor weights of the CDX mice in the Ctrl and *BAX*-OE groups. **(M)** Macroscopic appearance of tumors in the large intestine of AOM/DSS induced CRC model mice in the EV and *BAX-*OE groups (n = 10). **(N)** Kaplan-Meier curve of the AOM/DSS mice in the EV and *BAX*-OE groups (n = 10). The data are shown as the mean ± SD. *P < 0.05; **P < 0.01; ***P < 0.001.

To evaluate the functional conservation of non−mammalian BAX in human colorectal cancer (CRC) cells, we overexpressed the full-length *Xenopus laevis BAX* (NM_001085635) linked with a hemagglutinin (HA) tag in HCT116 and LOVO cell lines. RT−qPCR and Western blot confirmed robust transgene expression at mRNA and protein levels, respectively (n = 3, *P* < 0.01; **Figures 1A, B**). A Co-IP experiment via an HA antibody to purify BAX followed by Coomassie Brilliant Blue staining and mass spectrometry analysis, verified the presence and identity of the specific peptide from toad-derived BAX (PXD058360) (**Figures 1C, D**). Functional CCK-8 and colony formation assays showed that BAX overexpression (*BAX*-OE) significantly reduced CRC cell proliferation and colony-forming capacity compared to control empty-vector (EV) cells (n = 3, *P* < 0.05; **Figures 1E, F**). Annexin V-APC staining and WB of cleaved poly ADP ribose polymerase (cleaved PARP) and cleaved caspase 3 confirmed robust apoptosis induction (n = 3, *P* < 0.001; **Figures 1G, H**).

To validate the effect of BAX on CRC *in vivo*, the cell-derived xenograft (CDX) model was constructed by subcutaneous injection of HCT116 and *BAX*-OE cells into the right or left flanks of NOD-SCID mice, respectively. Tumors formed by *BAX*-OE cells exhibited slower growth and decreased endpoint tumor weight and volume (968.33 ± 90.15 *vs* 329.67 ± 30.59 mm^3^, 1.19 ± 0.20 *vs* 0.50 ± 0.10 g; n = 6, *P* < 0.01; **Figures 1I–L**). In the colorectum of AOM/DSS-induced CRC model mice, the *BAX-*OE group also revealed a marked deceleration of tumor growth (**Figure 1M**). Additionally, Kaplan-Meier survival curve analysis showed that the mice in *BAX*-OE group had significantly longer overall survival compared to EV group (n = 10, *P* = 0.0390; **Figure 1N**). These findings indicate that toad-derived BAX inhibits cell proliferation and promotes apoptosis in CRC.

### 3.2 Overexpressed BAX induces mitochondrial apoptosis via competitive human BCL2 binding

**Figure 2:**
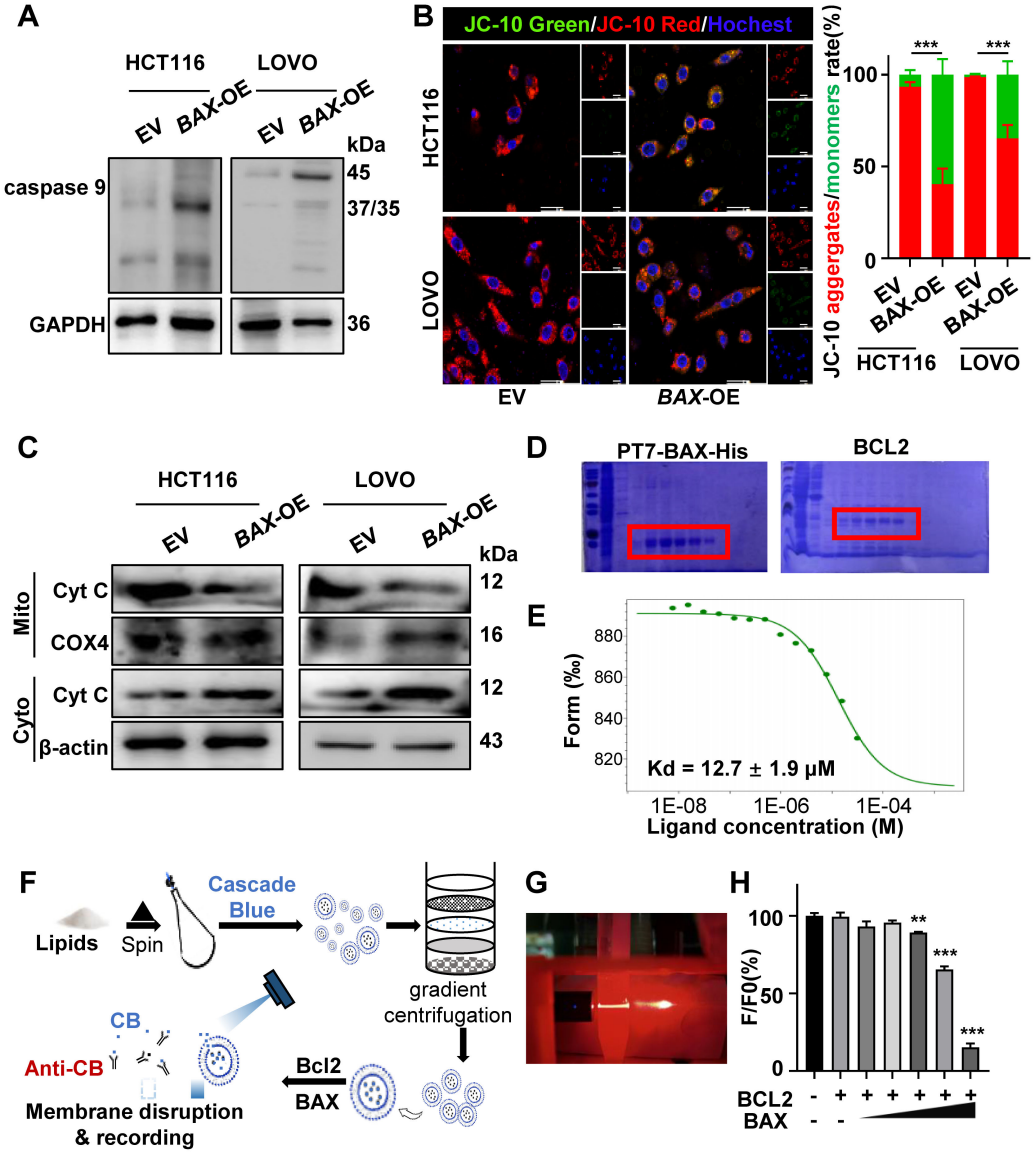
Overexpressed BAX induces mitochondrial apoptosis via competitively binding BCL2. **(A)** WB assay showed that overexpression of BAX increased caspase 9 expression. **(B)** JC-10 staining revealed that BAX overexpression induced mitochondrial outer membrane permeabilization, leading to the release of cytochrome c and subsequent apoptosis. **(C)** Subcellular localization analysis revealed that BAX overexpression significantly translocated cytochrome c from the mitochondria to the cytoplasm. **(D)** Expression and purification of His-tagged toad-derived BAX (PT7-BAX-His) and human BCL2 in *E. coli* BL21 (DE3). **(E)** MST experiments demonstrated the direct binding between toad-derived BAX and human BCL2 with a Kd of 12.7 ± 1.9 μM. **(F)** A schematic diagram showing that liposomes, mimicking mitochondrial membranes, were prepared using the film dispersion method and loaded with Cascade Blue prior to purification with gradient centrifugation. Membrane disruption by BAX/BCL2 was quantified via Cascade Blue release. **(G)** The Tyndall effect confirmed the uniformity of the liposomes. **(H)** The addition of BAX-His protein resulted in enhanced membrane disruption, particularly at higher concentrations. The data are shown as the mean ± SD. **P < 0.01; ***P < 0.001.

In the intrinsic pathway activation of apoptosis, BAX is a pro-apoptotic protein that binds to the anti-apoptotic protein BCL2, leading to the permeabilization of the mitochondrial outer membrane and subsequent release of Cyt *c*, triggering a cascade of events that culminates in apoptosis. We thus hypothesized that BAX could interact with BCL2 to induce cell apoptosis. The western blot revealed a significant upregulation of caspase 9 in *BAX*-OE CRC cells, indicating enhanced mitochondrial apoptotic initiation activity (**Figure 2A**). Furthermore, the JC-10 staining assay demonstrated more loss of mitochondrial outer membrane permeabilization (MOMP) in *BAX*-OE cells (*P* < 0.001; **Figure 2B**). Additionally, subcellular fraction analysis confirmed substantial translocation of Cyt *c* levels from mitochondria to the cytosol while increasing COX4 levels in the mitochondria (**Figure 2C**). This provides compelling evidence that BAX promotes MOMP and subsequent Cyt *c* release, thereby activating the intrinsic apoptotic pathway.

Importantly, the MST experiment using recombinant toad BAX and human BCL2 with His tag purified from *E. coli* validated a direct physical interaction between BAX and BCL2, yielding a dissociation constant (Kd) of 12.7 ± 1.9 μM (**Figures 2D, E**). Moreover, we prepared liposomes mimicking mitochondrial membranes via film dispersion and the encapsulation of Cascade Blue (**Figure 2F**). The uniformity of the liposomes was confirmed by the Tyndall effect (**Figure 2G**). Upon addition of BAX protein, we observed concentration-dependent membrane disruption (n = 3, **Figure 2H**), with co-addition of BCL2 attenuating permeabilization, consistent with competitive inhibition (3). These results suggest that toad BAX competes with pro-survival BCL2, thus inducing MOMP and downstream caspase activation to induce mitochondrial apoptosis.

### 3.3 Structural optimization of BAX strengthens the BAX-BCL2 interaction and augments anti-tumor activity

**Figure 3:**
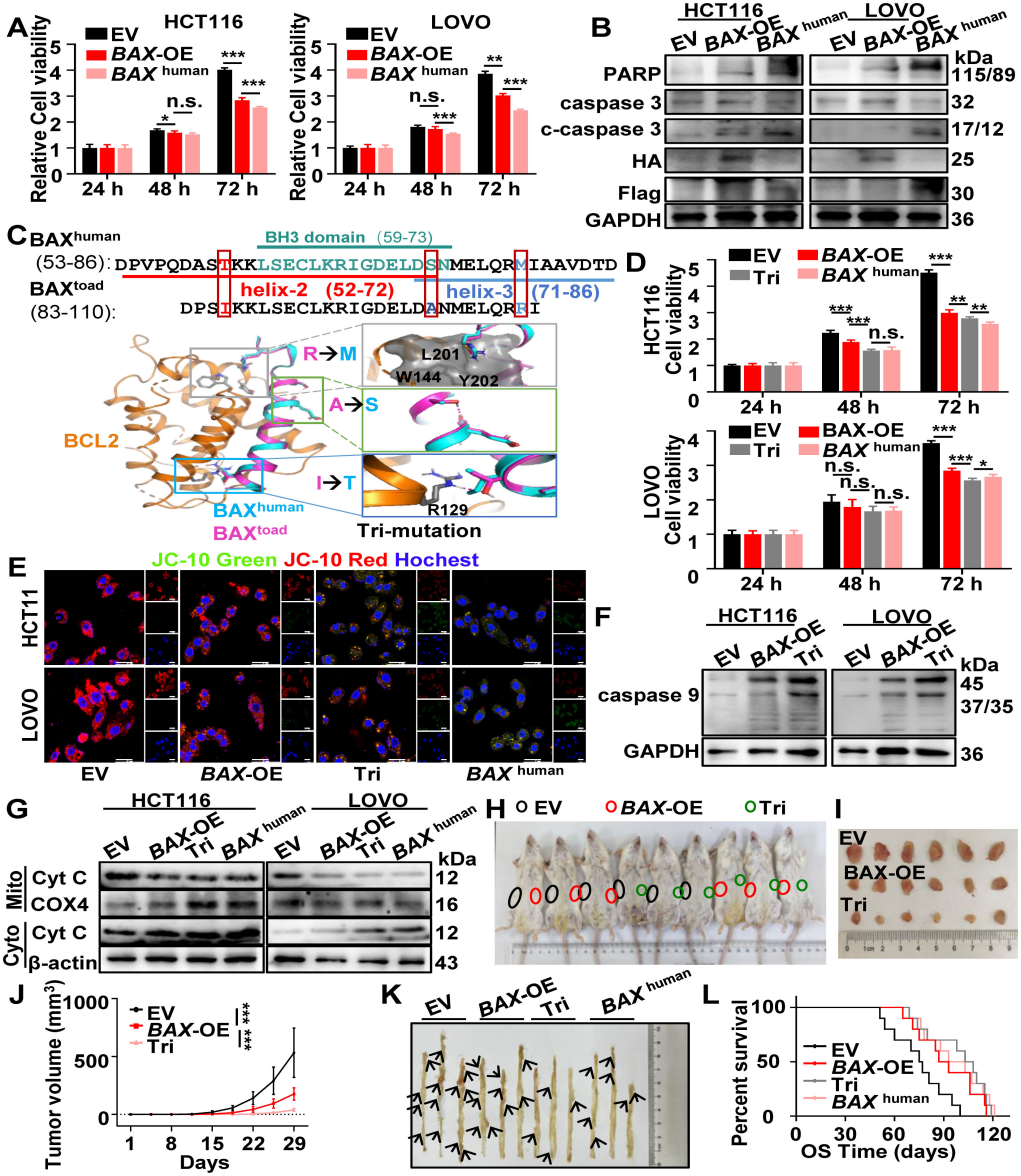
Structural optimization of BAX strengthens the interaction between BAX and BCL2. **(A)** Comparative analysis of human and toad BAX on inhibitory CRC cell viability via CCK8 assay. **(B)** WB analysis revealed that human BAX more effectively induced apoptosis in colorectal cancer cells, as evidenced by increased cleavage of PARP and caspase-3. **(C)** Molecular docking based on the Bcl-2 complex with Beclin 1 BH3 domain (PDB: 5VAU) identified and introduced three mutations (R to M, A to S, and I to T) in toad BAX to enhance its binding affinity with human BCL2. **(D)** CCK8 assays revealed that the triply mutated BAX (Tri) demonstrated an enhanced pro-apoptotic effect in CRC cells compared to non-mutated BAX, although its potency was slightly lower than that of human BAX. **(E)** JC-10 staining revealed that the triply mutated BAX induced a more pronounced loss of mitochondrial membrane potential in colorectal cancer cells, indicative of enhanced apoptosis. **(F)** WB analysis revealed increased levels of cytochrome c and caspase-9 in colorectal cancer cells overexpressing the triply mutated BAX, further confirming its enhanced apoptotic potential. **(G)** Subcellular protein quantification showed a significant translocation of cytochrome c from the mitochondria to the cytoplasm upon overexpression of triply mutated BAX, indicating its ability to induce mitochondrial outer membrane permeabilization and activate the intrinsic apoptotic pathway. **(H-J)** Tumors formed by triply mutated *BAX*-OE cells grew more slowly than those formed by WT and *BAX*-OE cells, exhibiting significantly reduced tumor volume. **(K)** Morphological observations demonstrated that elevated levels of triply mutated BAX markedly slowed tumor growth in the colorectum of AOM/DSS mice. **(L)** Kaplan-Meier curve of AOM/DSS mice revealed that the triply mutated *BAX*-OE group had significantly longer survival times compared to the EV or *BAX*-OE groups. The data are shown as the mean ± SD. *P < 0.05; **P < 0.01; ***P < 0.001, n.s. for no significant difference.

Although toad BAX induced apoptosis, its potency appeared inferior to that of human BAX, as evidenced by a comparative analysis of cell viability and cleavage of PARP and caspase-3 in both HCT116 and LOVO cells (n = 3, *P* < 0.05; **Figures 3A, B**), suggesting interspecies potency disparity (**Supplementary Figure 1**). To improve binding affinity, we subsequently utilized molecular docking based on the BCL2–Beclin1 BH3 domain complex structure (PDB: 5VAU), and introduced three mutations (I86T, A102S, R109M) to optimize the hydrophobic packing and hydrogen-bond networks at the BAX-BCL2 interface (**Figure 3C**). The resulting triple mutant (Tri−BAX) exhibited stronger inhibitory effects on cell viability, greater mitochondrial depolarization, and enhanced caspase-9 activation and Cyt *c* release compared to the non-mutated toad BAX, although its maximal cytotoxicity was slightly below human BAX (**Figures 3D–G**). Additionally, the cell apoptotic assay and the soft agar colony formation assay confirmedthe stronger inhibitory effects of the triply mutated BAX than the non-mutated version to induce apoptosis (**Supplementary Figures 2A, B**).

We further validate the anti-tumor effect of structurally optimized BAX *in vivo*. In the CDX model, these HCT116 WT, *BAX*-OE, and Tri-BAX cells were injected subcutaneously into the right or left flanks of NOD-SCID mice, respectively. Tumors formed by Tri-BAX group showed significantly slower tumor weight and volume (532.35 ± 194.39 *vs* 176.45 ± 48.20 *vs* 39.12 ± 11.35 mm^3^; n = 6, *P* < 0.001; **Figures 3H–J**; **Supplementary Figure 2C**). Moreover, AOM/DSS model mice in the Tri-BAX group had markedly longer survival time, with a marked reduction of tumor growth in the colorectum compared to mice in EV or *BAX*-OE groups (n = 10; **Figures 3K, L**). The mutations in BAX likely strengthen the BAX/BH3-in-groove interaction, amplifying BAX auto-activation and MOMP while competitively displacing endogenous BCL2 sequestration (3). Taken together, these results provide compelling evidence that Tri-BAX promotes mitochondrial outer membrane permeabilization and subsequent Cyt *c* release, via enhancing its interaction with BCL2, thereby strengthening its pro-apoptotic effects and improving survival outcomes in both *in vitro* and *in vivo* models.

### 3.4 Engineered exosomes enable the targeted delivery of functional BAX and effectively induce cytotoxicity *in vitro*

**Figure 4:**
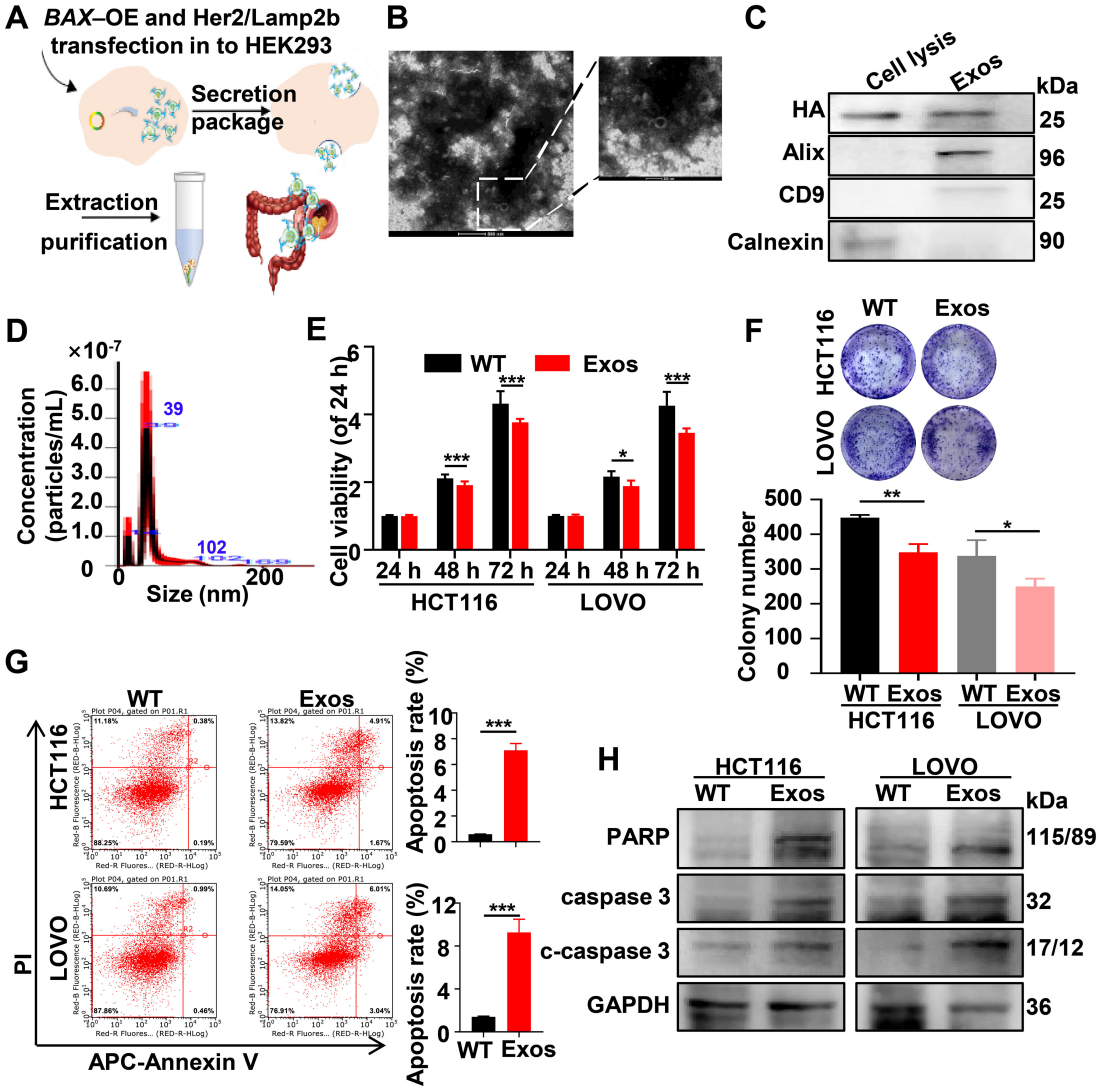
Engineered exosomes for targeted delivery of *BAX* effectively induce apoptosis *in vitro*. **(A)** Schematic diagram of the production and harvesting process of BAX EVs for targeted delivery of BAX. **(B)** Characterization of EVs using transmission electron microscopy (TEM). **(C)** WB analysis showed the presence of exosome markers Alix and CD9. **(D)** Nanoparticle tracking analysis (NTA) demonstrated that the average particle size of EVs is 39 nm. **(E)** CCK8 assay revealed that exosomes engineered to deliver BAX significantly inhibited the proliferation of CRC cells. **(F)** Colony formation assay revealed that exosomes engineered to deliver BAX significantly inhibited the proliferation of CRC cells. **(G)** Annexin V/PI staining apoptosis assay demonstrated that BAX induced apoptosis in CRC cells. **(H)** WB analysis revealed that human BAX effectively induced apoptosis in colorectal cancer cells, as confirmed by increased cleavage of PARP and caspase-3. The data are shown as the mean ± SD. *P < 0.05; **P < 0.01; ***P < 0.001.

To overcome delivery barriers, we engineered EVs/exosomes expressing BAX for targeted delivery to CRC cells. First, we transduced HEK293 cells with optimized Tri-BAX plasmids and then purified the resulting exosomes (**Figure 4A**). The nanoparticle tracking analysis (NTA) and transmission electron microscopy (TEM) confirmed the typical cup-shaped vesicles with a mean diameter of 39 nm, and WB analysis verified the expression of classic EVs markers, Alix and CD9 (**Figures 4B–D**). Furthermore, BAX−loaded exosomes (Exos) significantly inhibited proliferation, attenuated colony formation, and elevated apoptosis, evidenced by increased Annexin V/PI staining positive populations (n = 3, *P* < 0.05; **Figures 4E–G**). WB analysis of cleavage of PARP and caspase-3 also confirmed robust apoptosis induction by BAX (**Figure 4H**), recapitulating the direct overexpression phenotypes in CRC cells.

### 3.5 BAX-engineered exosomes suppress tumor growth with favorable safety in PDX and AOM/DSS CRC models *in vivo*

**Figure 5:**
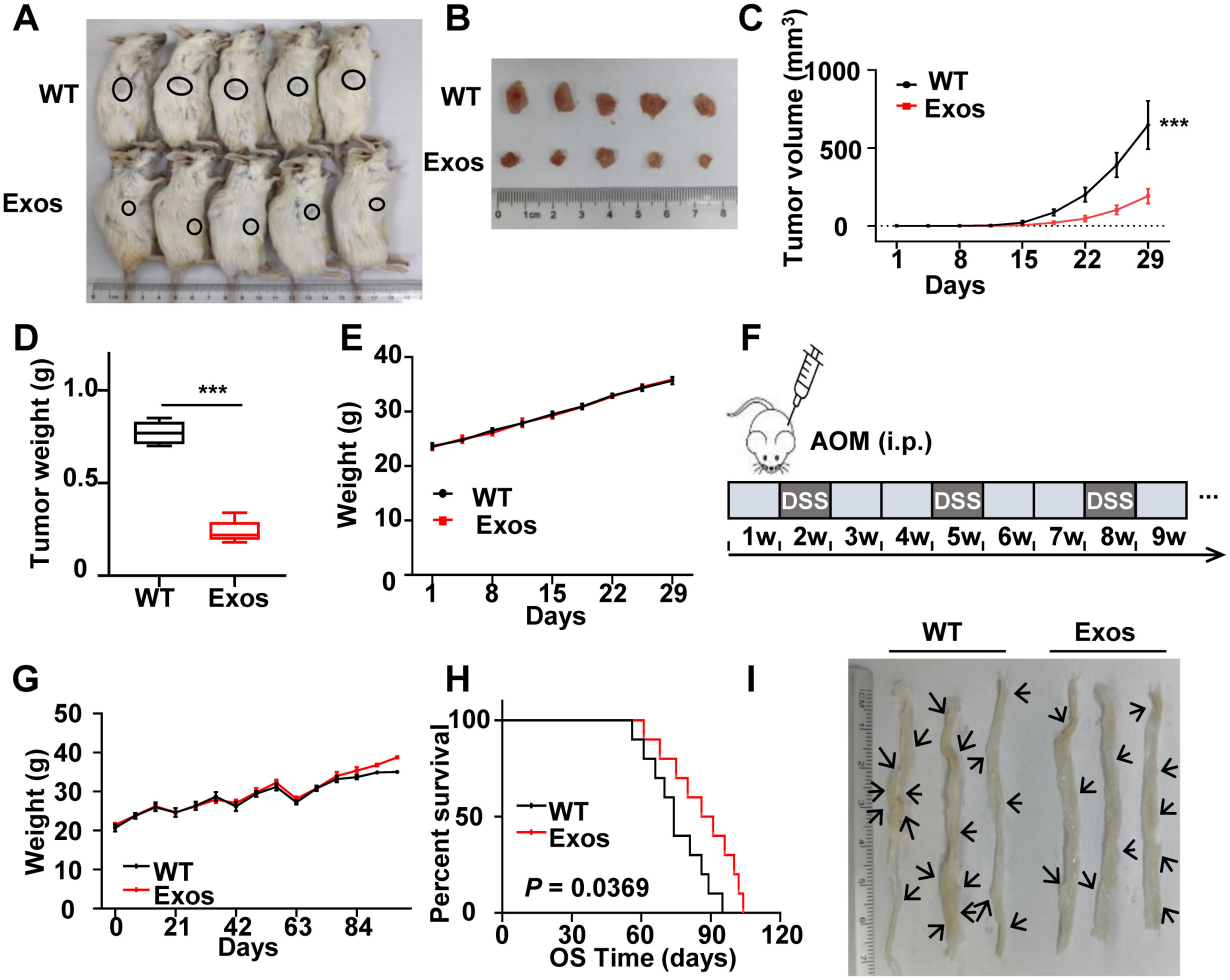
Engineered exosomes for targeted delivery of BAX effectively induce apoptosis in both patient-derived xenograft **(PDX)** and AOM/DSS induced CRC models *in vivo.* **(A, B)** Photographic images of PDX model mice on day 29 and tumors taken from mice in each group. **(C)** Tumor growth curves of the PDX mice in WT and *BAX*-EVs groups. **(D)** Tumor weights of the PDX mice in WT and *BAX-*EVs groups. **(E)** Body weight monitoring was used as a critical safety indicator, and the data confirmed that *BAX*-EVs did not induce adverse effects, highlighting their safety profile. **(F)** Scheme illustration of the AOM/DSS mouse model. **(G)** Body weight was closely monitored, reinforcing the safety of the *BAX*-EVs throughout the study. **(H)** Kaplan-Meier curve further validated that AOM/DSS mice in the *BAX*-EVs group exhibited significantly prolonged survival compared to those in the WT group. **(I)** Morphological evaluations visually confirmed that colorectal tumor growth in AOM/DSS mice was markedly reduced in the *BAX*-EVs group compared to the control group. The data are shown as the mean ± SD. ***P < 0.001.

To further validate the efficacy of BAX-EVs *in vivo*, we utilized a CRC patient−derived xenograft (PDX) model. The PDX model mice were administered intravenously normal saline or BAX-EVs (5 mg/kg) every other day. The tumors in BAX-EVs group had significantly lower average volume and weight compared to the control group (647.66 ± 138.84 *vs* 190.76 ± 42.40 mm^3^, 0.77 ± 0.06 *vs* 0.24 ± 0.05 g; n = 5, *P* < 0.001; **Figures 5A–D**). Moreover, body weight was monitored throughout the entire experiment, and the results underscore a favorable preliminary safety profile (**Figure 5E**).

In the AOM/DSS mouse model following a defined experimental scheme (**Figure 5F**), continuous body weight monitoring revealed no signs of treatment-related toxicity, reinforcing the therapeutic safety of BAX-EVs (**Figure 5G**). Importantly, systemic administration of the BAX-loaded exosomes had markedly extended survival time and visibly attenuated colorectal tumorigenesis (n = 10, *P* = 0.0369; **Figures 5H, I**). These results confirmed the ability of BAX-EVs to target CRC *in vivo*. Therefore, the exosome-delivered optimized BAX is both effective and well tolerated in these preclinical settings, highlighting its clinical translational potential.

## 4 Discussion

The present study provides the first evidence that a non−mammalian BAX ortholog can potently suppress CRC growth via direct engagement with the human mitochondrial apoptosis pathway, underscoring the deep evolutionary conservation of the BCL2-regulated cell death network across vertebrates (4, 13).

Although the pro−apoptotic function of BAX is evolutionarily conserved, we demonstrate that toad BAX not only retains this activity but also physically binds with human BCL2, as shown by microscale thermophoresis (Kd = 12.7 ± 1.9 μM) and liposomal disruption assays. The observation that exogenous BAX overexpression triggers apoptosis is consistent with the auto-activation model (3), wherein excess BAX molecules overwhelm endogenous BCL2 sequestration, enabling homotypic BAX oligomerization and pore formation to amplify apoptotic signaling and MOMP. These findings extend previous observations that BAX-BCL2 interactions are structurally plastic and can be modulated across species (6, 14).

A key insight from our work is that rational mutagenesis can bridge functional gaps between orthologs. Using a non-human starting template may help circumvent pre-existing auto-regulatory constraints that limit the activity of exogenously delivered human BAX. Toad BAX was chosen precisely because it exhibits lower basal activity than human BAX (**Figures 3A, B**). While toad wild-type BAX exhibits slightly suboptimal potency relative to human BAX, its robust *in vivo* activity indicates favorable pharmacokinetics or enhanced resistance to proteasomal clearance. The functional disparity between toad and human BAX, far from being a mere disadvantage, offers a valuable structure-function gradient for affinity engineering. Importantly, the triple mutation strategy (Tri-BAX), designed to increase binding to the hydrophobic BAX/BCL2 groove, successfully narrowed this gap while maintaining satisfactory compatibility with exosome encapsulation and anti-CRC efficacy. Therefore, the optimization framework starting with a weaker binder facilitates to reduce the risk of unwanted constitutive activity during protein production and exosome loading, while allowing activation at the target site. Since one such homolog can be functionally “humanized” via minimal mutation, it sets and expands a new filed of mining this diversity for therapeutic leads. This approach aligns with well-established principles from BH3 peptide engineering with side-chain substitutions (15), highlighting a pragmatic structure-function balance. In contrast to small molecules or BH3 mimetics that indirectly release endogenous BAX by targeting anti-apoptotic pockets (10), direct delivery of BAX protein simultaneously titrates BCL2 and executes MOMP, bypassing upstream signaling requirements. Additionally, these two modes of action, de-repression or direct effector delivery, are complementary and may be combined to overcome resistance mechanisms that limit BH3 mimetic efficacy in solid tumors.

Therapeutic proteins face intrinsic pharmaceutical challenges, including poor stability, limited cell entry, and potential immunogenicity (16). EVs and exosome-based delivery provide a biologically native vehicle that preserves protein conformation and possesses intrinsic membrane fusion ability, thereby facilitating cytosolic cargo release and tumor homing via intrinsic tropism (17). Here, engineered exosomes, harboring canonical markers (CD9/Alix-positive) appropriate size (~39 nm) and cargo integrity, served as a safe and effective vehicle. Unlike viral systems, exosomes did not induce weight loss or overt toxicity in mice, consistent with their low immunogenicity (18). Moreover, BAX−loaded exosomes suppressed tumor growth in PDX models, which recapitulate human tumor heterogeneity more accurately than CDX models. These results position exosomal BAX as a promising off-the-shelf biologic, consistent with over 200 registered clinical trials evaluating exosome-based therapies (19).

Several aspects warrant further investigation. First, the precise stoichiometry and dynamics of suboptimal BAX activation remain to be elucidated, potentially via deep mutational scanning or computational affinity improvement. Second, cross-species immunogenicity, long-term tissue retention, and pharmacokinetic profiling remain to be quantified. Third, future work should integrate tumor-targeting ligands (HER2/EGFR peptides) and combine BAX−exosomes with existing chemotherapies, immune checkpoint inhibitors, or BH3 mimetics (to lower BCL2 levels) to achieve synergistic effects, given that apoptosis induction can enhance immunogenic cell death.

From a broader perspective, this work presents a proof-of-concept framework that may establish a promising, generalizable approach: leveraging evolutionary diversity to characterize function-distinct proteins, optimizing them via structure-guided engineering, and delivering them through endogenous vesicle systems. This strategy might be extended to other challenging, undruggable intracellular targets in oncology and beyond.

In conclusion, this study demonstrates that toad−derived BAX, when rationally optimized and exosomally delivered, can directly reactivate the mitochondrial apoptosis pathway as a pro-apoptotic agent to effectively and safely suppress CRC tumor growth. It not only develops pro-apoptotic protein biologics, from the identification of a natural cross-species effector via mechanistic dissection and rational protein engineering to targeted biocarrier delivery, but also provides pre-clinical protein-based therapeutics with potential applicability to other resistance or apoptosis-refractory solid malignancies.

## Data Availability

The MS data (PXD058360) have been deposited at GEO or ProteomeXchange Consortium and are publicly available as of the publication date. All data that support the findings of this study are available from the corresponding authors upon reasonable request.

## Supplementary material

Supplementary Figure 1 Multiple sequence alignment of BAX orthologs from *Xenopus laevis* (NP_001079104.1), *Homo sapiens* (NP_001278357.1) and *Mus musculus* (NP_031553.1). Three moderately conserved sites (I/T, A/S, and R/M) inside or outside of BH3 domain are highlighted in red rectangle.

Supplementary Figure 2 Structural optimization of BAX enhanced its anti-CRC effect. **(A)** Apoptosis assay revealed an enhanced apoptotic rate in single R109M-mutated or triply mutated *BAX*-OE cells compared to EV cells. **(B)** Colony formation assay demonstrated that triply mutated *BAX*-OE cells exhibited reduced colony formation compared to EV cells. **(C)** Tumors formed by triply mutated *BAX*-OE cells showed significantly reduced growth rates and tumor volumes compared to those formed by EV or *BAX*-OE cells. The data are shown as the mean ± SD. **P* < 0.05; ***P* < 0.01; ****P* < 0.001.
