# Supplementary material for: Engineered exosomes deliver structurally optimized toad BAX to reactivate mitochondrial apoptosis in colorectal cancer
Source: Front Oncol. 2026 Jun 24;16:1882315. doi: 10.3389/fonc.2026.1882315 (PMC13341481; doi:10.3389/fonc.2026.1882315)
Supplement: Supplementary Figure 1 — Multiple sequence alignment of BAX orthologs from Xenopus laevis (NP_001079104.1), Homo sapiens (NP_001278357.1) and Mus musculus (NP_031553.1). Three moderately conserved sites (I/T, A/S, and R/M) inside or outside of BH3 domain are highlighted in red rectangle. [file DataSheet1.docx]

Supplementary Material


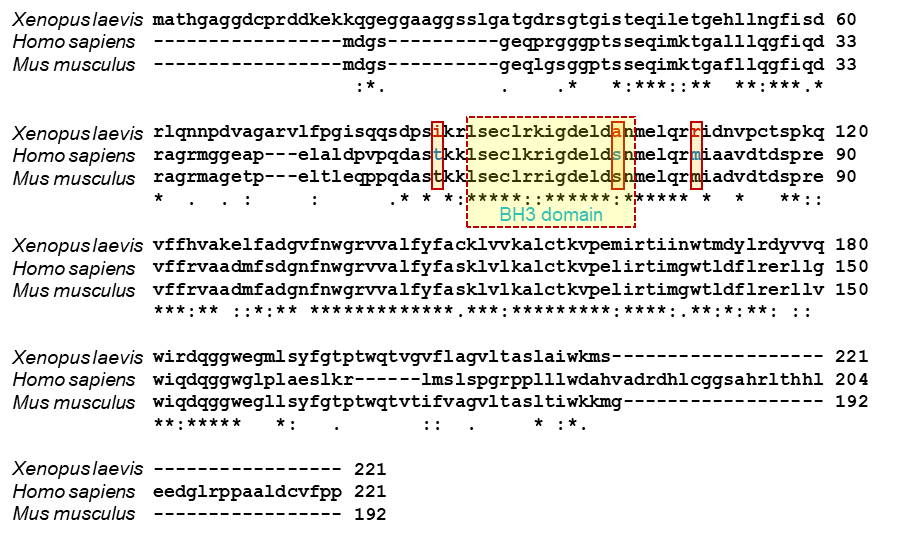


**Figure S1 Multiple sequence alignment of BAX orthologs from Xenopus laevis (NP_001079104.1), Homo sapiens (NP_001278357.1) and Mus musculus (NP_031553.1).** Three moderately conserved sites (I/T, A/S, and R/M) inside or outside of BH3 domain are highlighted in red rectangle.

**
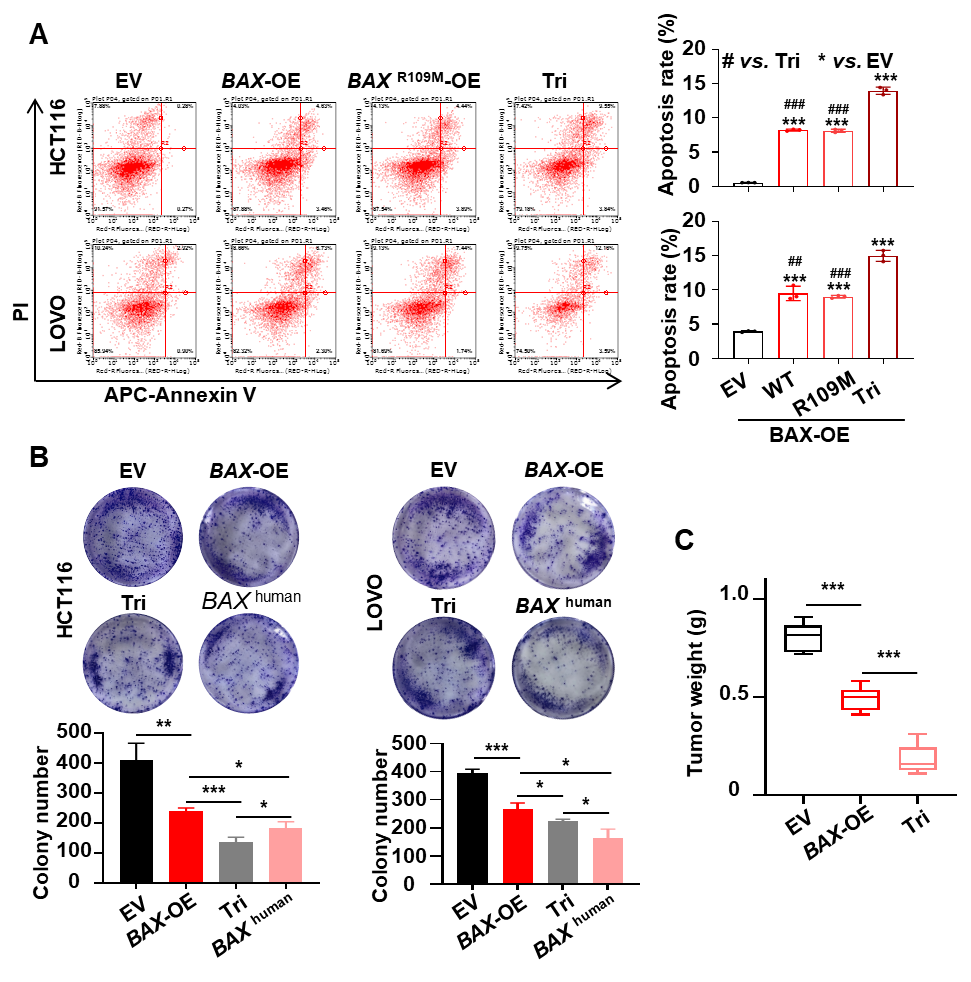
**

**Figure S2 Structural optimization of BAX enhanced its anti-CRC effect.** (A) Apoptosis assay revealed a higher rate of apoptosis in single R109M mutated or triply mutated *BAX*-OE cells compared to EV cells. (B) Colony formation assay demonstrated that triply mutated *BAX*-OE cells exhibited reduced colony formation compared to EV cells. (C) Tumors formed by triply mutated *BAX*-OE cells showed significantly reduced growth rates and tumor volumes compared to those formed by EV or *BAX*-OE cells. Data are shown as the mean ± SD. **P* < 0.05; ***P* < 0.01; ****P* < 0.001.
